# Radiological assessment of periodontal ligament space visibility on third molars for forensic age assessment — a comparison study of three different staging scales

**DOI:** 10.1007/s00414-024-03184-5

**Published:** 2024-02-17

**Authors:** Maximilian Timme, Laurin Steffens, Jan Viktorov, Adam Streeter, André Karch, Andreas Schmeling

**Affiliations:** 1 Institute of Legal Medicine, University Hospital Münster, Röntgenstraße 23, 48149 Munster, Germany; 2 Institute of Epidemiology and Social Medicine, University of Münster, Domagkstraße 3, 48149 Munster, Germany

**Keywords:** Age estimation, Dental age, Degenerative characteristics, Third molar, Orthopantomogram

## Abstract

Various staging scales have been proposed for the assessment of the visibility of the periodontal ligament space of mandibular third molars on dental panoramic radiographs (PANs) for forensic age assessment in living individuals. However, up to now, there has been no systematic comparison between these staging scales available. We directly compared the 2010 staging scale proposed by Olze et al. with the 2017 staging by Lucas et al. and the 2020 staging by Guo et al. in a German study population. We evaluated 233 PANs from 115 females and 118 males aged 20.0 to 40.9 years using three independent examiners, with one examiner conducting two assessments. We examined the correlation between age and stage, as well as the inter- and intra-rater reliabilities. While the point estimates for the correlation coefficient and the reliability measures were lowest for the Guo scale and highest for the Olze scale, confidence intervals showed a large overlap, particularly for the scales of Olze et al. and Lucas et al. The correlation coefficients between stage and age were consistently lower in females than in males across all methods. In summary, we showed that the staging scales of Olze et al. and Lucas et al. were very similar. The Olze method showed higher point estimates across all analyses, and because there are more reference data available for this method, we argue that it should be preferred as the method of choice for further studies in the field. However, Guo method could be considered for instances, in which the inter-radicular periodontal ligament is not evaluable.

## Introduction

In the field of forensic sciences, the determination of chronological age via age assessment procedures is a well-established practice [1–3]. The accuracy of age determination is vital for legal proceedings and administrative actions, especially for unaccompanied minor refugees [4–7]. This is due to the necessity of establishing age with the highest level of certainty concerning important legal thresholds [1]. With the increasing movements of transborder migration and the associated rise in individuals lacking clear age information, forensic age assessment will continue to be an area of interest in the future [7, 8]. Therefore, forensic age assessment has become an active area of research within forensic sciences [9–18].

The Study Group on Forensic Age Diagnostics recommends the inclusion of dental status examinations in age assessments [19]. For this purpose, a dental panoramic radiograph (PAN) is usually conducted [19]. Typically, the mineralization and eruption of third molars are assessed and compared to reference populations [20–22]. However, the completion of tooth development, including that of the third molars, can occur prior to the age of 18, which is the age of majority in many legal systems and thus a forensically crucial age limit [23]. As a result, it is not always feasible to make decisions about an individual’s age relative to this threshold with the required degree of certainty, based solely on tooth development features.

Following the completion of tooth development, degenerative tooth characteristics can be employed for age assessment [24–30]. However, degenerative tooth characteristics are more susceptible to external factors such as diet, habits, medication, or disease than tooth development features, which are primarily genetically determined [31, 32]. Hence, a comprehensive examination is required in each case to differentiate between age-related degeneration and pathological conditions, in order to determine the viability of evaluating the teeth in question [33–35]. In order to ensure the accuracy and reliability of the results, age assessment based on pathological teeth should be avoided.

Over time, various degenerative tooth characteristics have been identified as potential indicators for chronological age. One such characteristic is the reduction in the visibility of the periodontal ligament space of mandibular third molars on PAN [28]. There have been various studies suggesting that this characteristic can be used for forensic age assessment [28, 36–39], although it has not been conclusively determined whether the feature is a purely radiographic phenomenon or whether the periodontal ligament literally degenerates. The periodontal ligament as the “tooth retaining structure” is the connection between the tooth and the jaw. Collagen is the primary protein found in the periodontal ligament, while fibroblasts are the predominant cells present in this tissue. Known age-related changes in the periodontal ligament include a decrease in the density of fibroblasts. In addition, a general decrease in cellular elements has been described [40–42]. Due to the necessity of a relatively flexible suspension of the tooth in the jaw during loading, an ossification of the periodontal gap is not physiologically predetermined. Rather, S100A4, a member of the S100 calcium binding protein family, regulates the expression of osteoblastic genes and thus prevents mineralization of the periodontal ligament [43]. It has been known for a long time that the function, i.e., the loading of a tooth, has an impact on the thickness of the periodontal gap and on the cementum apposition [44–47]. Putting this evidence together, it is most likely that a variety of age-associated changes in the periodontal gap and in the surrounding bone cause a corresponding visual effect in the summation radiology.

Our study aimed to directly compare three proposed stage classifications for assessing the decrease in radiographic periodontal ligament visibility on mandibular third molars [28, 48, 49] in the age group from 20 to 40 years with respect to inter- and intra-rater reliability as well as correlation with biological age.

## Material and method

Our study is based on digital dental panoramic radiographs (PANs) obtained from a university dental clinic located in the North Rhine-Westphalia region of Germany. All X-ray images were taken for medical indications. Data were anonymized before evaluation so that it was not possible to trace them back to individuals. The study population comprised patients from dental surgery, orthodontics, prosthodontic, and conservative dentistry departments. The participants’ chronological ages at the time of the radiographs were collected by presenting the appropriate official documents, usually German insurance identification cards. Radiographs were evaluated using the synedra View Personal software version 22.0.0.1 (synedra information technologies GmbH, Innsbruck, Austria) in DICOM format, with examiners using the software’s magnification and gray level adjustment tools. The technical equipment and the ambient light conditions were identical for all examiners. Three board-certified dentists were responsible for conducting the evaluations.

The sample size for the present study was determined by referring to comparable studies in the literature [50, 51]. Consequently, a total sample size of 200 digital panoramic radiographs was aimed for. In order to compensate for later exclusions, a total of 300 radiographs were initially collected.

The inclusion criteria mandated that images be of sufficient quality to facilitate radiological detection of teeth 38 [FDI] and 48, which were required to be free of caries or restorations. In addition, third molars had to exhibit stage H of development, as per the Demirjian et al. classification [21], which corresponds to a completed development. Participants with any genetic disorder or jaw-related diseases were excluded from the study. To evaluate intra-examiner reliability, one examiner reassessed the entire dataset.

The assessments were executed based on the ensuing stage classifications:

Olze et al. (2010) (“Olze”) [28]

- Stage 0 = The periodontal ligament space is visible along the full length of all roots.
- Stage 1 = The periodontal ligament space is invisible in one root from apex to more than half root.
- Stage 2 = The periodontal ligament space is invisible along almost the full length of one root or along part of the root in two roots or both.
- Stage 3 = The periodontal ligament space is visible along almost the full length of two roots.

Lucas et al. (2017) (“Lucas”) [49]

- PLV-A: 100 to 74% of the periodontal ligament space around the lower left third molar is discernible on the PAN.
- PLV-B: 75 to 50% of the periodontal ligament space is visible.
- PLV-C: 50 to 25% of the periodontal ligament space of the lower left third is visible when summated across the mesial and distal roots.
- PLV-D: 25 to 0% of the periodontal ligament space is discernible.

(PLV = periodontal ligament visibility)

Guo et al. (2020) (“Guo”) [48]

(Only the outer parts of lower third molar roots (mesial part of the mesial root and distal part of the distal root) are evaluated)

- Stage 0 = The periodontal ligament space is visible along the full length of all roots.
- Stage 1 = The periodontal ligament space is invisible in one root from apex to more than half root.
- Stage 2 = The periodontal ligament space is invisible along almost the entire length of one root or along part of the root in two roots.
- Stage 3 = The periodontal ligament space is invisible along almost the entire length of two roots.

Before starting with the actual evaluation of the X-ray images, the examiners underwent a calibration process to minimize potential biases due to variations in their experience with the methodology. The calibration involved the evaluation of 50 randomly selected images that were not part of the main dataset by each examiner. Any discrepancies in the assessment of the images by the examiners were discussed, and a consensus was reached for images that showed differences of more than one stage.

Data management and statistical analyses were performed in Stata, version 13.0 (Stata Corp LP, College Station, TX, USA). Tooth 38 and 48 staging was investigated as means of potentially classifying persons of unknown age into age groups. Following classification by the three raters, according to each method, the distribution of ages was subsequently compared across the stages of each method. Spearman’s rank correlation coefficient evaluated the correlation between age and stage. Age was then regressed on stage for each method and sex, adjusting for tooth. The degree to which rating might explain the variation in age was assessed through the adjusted coefficient of determination (adj-*R*^2^) and the specific proportion of variance explained (*ω*^2^) by rating. Krippendorff’s alpha (*α*) was used to evaluate agreement between and within raters. Fleiss’ kappa was also calculated. Repeatability of one rater and the reproducibility of all three raters were investigated for each method as means of evaluating the reliability of each method.

## Results

After exclusion of 67 radiographs due to detectable diseases of the teeth or bone, incomplete development of the mandibular third molars, or orthodontic appliances on the teeth, 233 PANs with a theoretical maximum of 466 teeth to be evaluated from 115 females and 118 males aged between 20.0 and 40.9 years were eligible for the present study (Table 1).

**Table 1:** Age and sex distribution of the study population

Age	Male (*n*)	Female (*n*)
20	4	7
21	8	6
22	6	8
23	11	9
24	4	7
25	14	4
26	5	6
27	4	5
28	4	4
29	4	4
30	4	4
31	5	6
32	6	5
33	5	5
34	4	5
35	5	4
36	5	5
37	5	5
38	5	7
39	5	4
40	5	5
Total (*n*)	118	115

In the evaluations, 49 teeth (10.52%) were found to be non-evaluable for the Olze method. For the Guo and Lucas methods, these values were 39 (8.37%) and 45 (9.66%) teeth, respectively (Table 2). The main reason for the non-evaluability was insufficient image quality in the area of interest. The superimposition of structures and blurring that are typically found in PANs were key reasons for their exclusion. Non-evaluability of the intra-radicular region often resulted from even slight rotations of the tooth. Overall, the method proposed by Guo et al. enabled the assessment of the highest number of teeth.

**Table 2:** Number of teeth that could not be evaluated depending on the staging method

	Tooth		Total (*n*)
Method	FDI 38	FDI 48	
Olze	26	23	49
Guo	22	17	39
Lucas	24	21	45

All stages could be identified in the three classifications evaluated. For the correlation between stage and age, the Spearman correlation coefficients for males were *ρ* = 0.362 (95% confidence interval 0.303, 0.425) and *ρ* = 0.215 (95% CI 0.152, 0.281) for females, using the Olze method. For the Lucas method, the Spearman correlation coefficients were *ρ* = 0.317 (95% CI 0.255, 0.376) and *ρ* = 0.170 (95% CI 0.108, 0.236), for males and females, respectively. For the Guo method, these were *ρ* = 0.312 (95% CI 0.250, 0.370) and *ρ* = 0.166 (95% CI 0.099, 0.230), for males and females, respectively (Table 3). The *ω*^2^ values confirm these findings. The confidence intervals for the results of all three methods overlap, but the values obtained for females are lower than those obtained for males (Table 3).

**Table 3:** Spearman’s correlation coefficients between stage and age for each method and sex with the adjusted *R*^2^ coefficient and partial omega-squared (*ω*^2^) value for the stage from the regression of age on stage for each method and sex, adjusted for tooth (95% confidence intervals)

Sex	Method	Spearman’s *ρ* (95% CI)	Adjusted *R*^*2*^	Partial *ω*^2^ (95% CI) for stage, adjusted for tooth
Males	Olze	0.362 (0.303, 0.425)	0.205	0.206 (0.159, 0.248)
	Guo	0.312 (0.250, 0.370)	0.114	0.115 (0.076, 0.152)
	Lucas	0.317 (0.255, 0.376)	0.142	0.143 (0.100, 0.182)
Females	Olze	0.215 (0.152, 0.281)	0.054	0.055 (0.026, 0.03)
	Guo	0.166 (0.099, 0.230)	0.081	0.082 (0.047, 0.113)
	Lucas	0.170 (0.108, 0.236)	0.027	0.028 (0.007, 0.050)

The intra-rater reliability coefficients were highest among the males, ranging from a Krippendorff alpha (*α*) of 0.565 (95% CI 0.484, 0.646) for the Guo method to 0.678 (95% CI 0.608, 0.748) for the Olze method (Table 4). While there was considerable overlap in the confidence intervals of the intra-rater coefficients in males, there was a significant difference in the intra-rater reliability coefficients for females, which ranged from a *α* of 0.363 (95% CI 0.279, 0.446) for Guo to 0.649 (95% CI 0.571, 0.727) for the Olze method (Table 4).

**Table 4:** Intra-rater reliability for each method applied to both sexes combined. *k* Fleiss’ kappa. *α* Krippendorff’s alpha (95% confidence intervals)

Sex	Method	*κ* (95%CI)	*α* (95%CI)
Males	Olze	0.641 (0.562, 0.720)	0.678 (0.608, 0.748)
	Guo	0.580 (0.503, 0.656)	0.565 (0.484, 0.646)
	Lucas	0.610 (0.535, 0.685)	0.620 (0.546, 0.694)
Females	Olze	0.648 (0.570, 0.726)	0.649 (0.571, 0.727)
	Guo	0.361 (0.278, 0.445)	0.363 (0.279, 0.446)
	Lucas	0.489 (0.409, 0.568)	0.490 (0.410, 0.569)

Inter-rater reliability was found to be highest using the Olze method, achieving an *α* of 0.558 (95%CI 0.489, 0.626) in males and 0.526 (95%CI 0.461, 0.590) in females, followed by the Lucas method (*α* = 0.546 (95%CI 0.480, 0.612), males and *α* = 0.479 (95%CI 0.4414, 0.543), females) and then the Guo method (*α* = 0.519 (95%CI 0.459, 0.580) in males and *α* = 0.428 (95%CI 0.364, 0.495) in females). While there was a clear positive trend in inter-rater reliability from Guo to Olze, these differences were not significant, given the overlapping confidence intervals for these estimates (Table 5). The same conclusions could be drawn from the Fleiss’ kappa coefficients that were also reported.

**Table 5:** Inter-rater reliability for each method applied to both sexes. *k* Fleiss’ kappa. *α* Krippendorff’s alpha (95% confidence intervals)

Sex	Method	κ (95%CI)	α (95% CI)
Males	Olze	0.557 (0.489, 0.625)	0.558 (0.489, 0.626)
	Guo	0.501 (0.437, 0.566)	0.519 (0.459, 0.580)
	Lucas	0.545 (0.479, 0.612)	0.546 (0.480, 0.612)
Females	Olze	0.525 (0.461, 0.590)	0.526 (0.461, 0.590)
	Guo	0.427 (0.364, 0.491)	0.428 (0.364, 0.492)
	Lucas	0.478 (0.413, 0.543)	0.479 (0.414, 0.543)

## Discussion

In this study comparing staging scales for assessing the visibility of the periodontal ligament, correlation with age was highest for the Olze scale, followed by the scales of Lucas et al. and Guo et al. However, the relatively small differences between the methods reflect their similarities, especially when comparing the methods of Olze and Lucas. There was considerable overlap in the confidence intervals for the point estimates, which were nearly equivalent for the methods of Lucas et al. and Guo et al. The differences in rating examiner agreements between methods and sexes were small overall, except for the intra-rater agreement results in females, where the Olze method performed significantly better than the Lucas and Guo methods. Nevertheless, the Olze method consistently achieved the highest point estimates for examiner agreement across all tests.

Our study was designed as a comparative study between the three evaluated staging scales and was not intended to serve as a reference study for the characteristic of periodontal ligament visibility in PANs. Therefore, the criteria for reference studies in age assessment did not need to be considered [52]. Consequently, descriptive measures for individual stages could also be omitted. Possible bias due to the study population or the study design would have affected all three methods equally.

Fundamentally, the periodontal ligament visibility is often challenging to determine, especially in higher stages. In the lower stages, for instance, it reveals greater clarity when it is possible to trace the periodontal ligament around the entire tooth. In the application of a morphological staging scale, a degree of subjectivity on the part of individual examiners invariably persists. These difficulties were reflected in the examiner agreements. Due to the low level of agreements, particularly in the inter-rater data analysis, it must be assumed that “incorrect” stage assignments were made which in turn distort the values for the correlation with age.

Chaudhary and Liversidge in 2017 have already named different root morphologies that complicate the evaluation of periodontal ligament visibility on third molars [53]. This involves the following findings: root apices in close proximity, root apices overlapping, apical third of roots in close proximity, and mesial root curved and out of focal trough [53].

To date, limited information is available in the literature on the general correlation of periodontal ligament degeneration with age. In 2014, Sequeira et al. published Spearman’s rho values for the method of Olze et al. in a Portuguese population. They had studied a total of 259 females and 228 males aged 17 to 31 years. The published values were *ρ* = 0.607 for the females and *ρ* = 0.400 for the males [54]. Thus, they were able to demonstrate a strong correlation in females and a moderate correlation in males [55]. In males, our study also found a moderate correlation across all methods, whereas in females our correlation coefficient was considerably lower [55, 56]. General physiological studies failed to detect any sex difference [57]. Future studies should further investigate a possible sex difference in the feature of radiographic periodontal ligament degeneration. The reason for the differences between the results of our study and those of Sequeira et al. is most likely to be found in the characteristics of the different study population and potential biases in that. One potential cause could lie in the differing age ranges of the study populations. Sequeira et al., for instance, examined younger individuals, specifically including those younger than 18 years. It is conceivable that periodontal ligament visibility in PAN may behave differently in younger individuals, exhibiting a larger correlation with age.

Additionally, one must consider the fundamental influence of external factors on degenerative dental features. These factors accumulate with advancing age, necessitating consideration. Therefore, it is not improbable that the correlation with age in degenerative dental features could fundamentally decrease with higher age. This should be clarified in the future through appropriately designed studies.

In 2021, Shah and Angadi examined a total of 339 PANs (180 males, 159 females) from age 15 to 40 years in an Indian study population using the Olze method. They found a correlation coefficient of *r* = 0.717 for tooth 38 and *r* = 0.714 for tooth 48 without having separated the sexes [39]. In this study as well, younger individuals were included, which could account for the higher correlation with age in comparison to our study.

Overall, reference studies in different ethnic groups are urgently needed in the future to further investigate the correlation of radiographic periodontal ligament degeneration with chronological age. Particularly, the influence of age on the extent of the correlation should be examined.

**Fig. 1:**
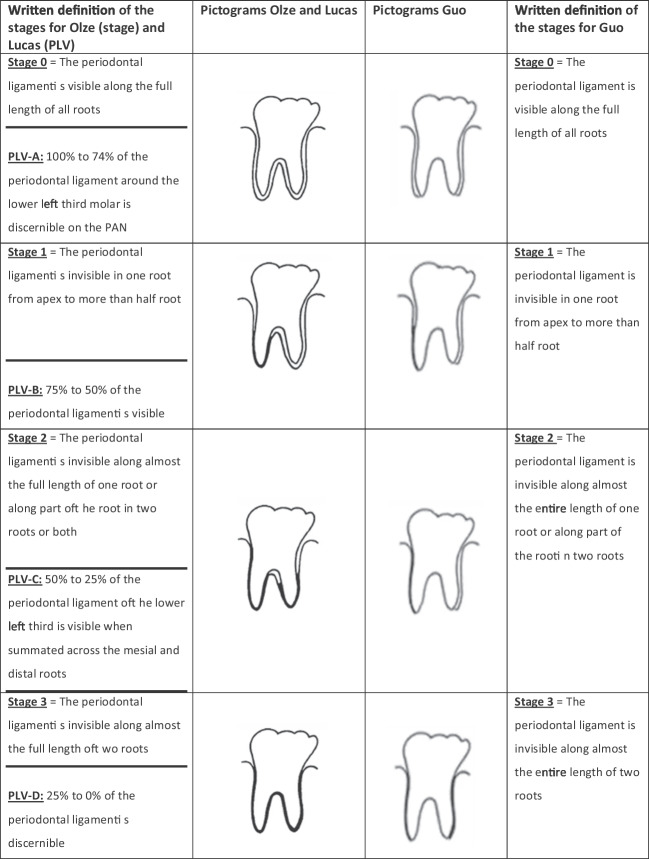
Stagings according to Olze et al., Lucas et al., and Guo et al. [28, 48, 49]. The pictograms for the methods of Olze et al. and Lucas et al. are identical

The difference between the classifications of Olze et al. and Guo et al. becomes clear when looking at the corresponding pictograms (Fig. 1). In the classification according to Guo et al., the inter-radicular region is not taken into account; rather, only the mesial and distal periodontal ligament is evaluated. This is important because the text description for the stages is identical for the Olze and Guo methods.

In contrast, the pictograms of the stages for the Lucas et al. method are largely identical to those of Olze et al. [28, 49] (Fig. 1). The minimal differences between the original pictograms, which do not affect the content, are most likely due to copyright reasons. For this reason, we have not included the pictograms from Lucas et al. in Fig. 1.

The slightly reduced correlation with age of the Guo method in the direct comparison can potentially be explained as this method does not take into account the periodontal ligament between the roots, which may result in the loss of age-dependent information. Another reason may be due to our study population. The Guo method was specially developed for Asian (Chinese) populations, with the assumption that the shape of the third molars could be different to other ethnic groups, particularly with a higher prevalence of fused or closely positioned roots [37, 48]. Such cases would have been regularly unclassifiable by the Olze method according to its stage definitions [37]. As a consequence, 10 more teeth could be evaluated with the Guo method compared to the Olze method (Table 2). However, the overall difference in the evaluability was only around two percentage points. Whether this effect is amplified in other populations or ethnicities needs to be investigated in future studies. Moreover, future comparative studies need to clarify whether the Guo method is superior in targeted cohorts and whether the correlation with age is better in these cohorts. A first validation study in a South Indian population from 2021 by Ray et al. reports correlations of 0.455 to 0.555 for the Guo method [58]. Overall, the use of the Guo method can be considered a viable alternative in cases where the inter-radicular periodontal ligament is not evaluable, such as it is the case with fused roots which preclude the use of the Olze or Lucas method.

The difference in the number of non-evaluable teeth between the Olze and Lucas methods seems surprising at first, as these cases should basically be congruent. However, it must be recognized here that the teeth were evaluated independently of each other and separately for each staging scale. The difference in the number of non-evaluable teeth between the Olze and Lucas methods must have arisen because of rater differences. Therefore, in borderline cases, a tooth may have been assessed as analyzable for the Olze method but not for the Lucas method. An example of this would be overlay effects that create a milky glass effect over the region of interest. These overlay effects are inherent to the PAN, but the transition to non-analyzability is gradual. All in all, this effect is reflected in the examiner agreement values.

The Lucas method showed lower point estimates for inter- and intra-rater agreement compared to the Olze method. This might be the case because of how the Lucas method is based on a continuous scale, the use of which is unlikely to distinguish between 74 and 75% visibility of the periodontal ligament. We also assume that differences of over 10% are only reproducible to a limited extent.

On the other hand, it is unclear why the Guo method did not perform better, given the restriction to the mesial and distal periodontal ligaments should have simplified the assessments overall. Reliability was also expected to be higher, because Guo et al. had presented considerably higher kappa coefficient values for the intra-examiner agreement of 0.843 and the inter-examiner agreement of 0.788 in their study compared to ours [48]. Regarding our results, it should be noted that the Guo method achieved the lowest values not only for the intra-rater, but also for the inter-rater agreement. Thus, the Guo method suffered from poor repeatability and reproducibility in its application to our study population. Currently, there are too few studies on this method in the literature to verify these results. Although Ray et al. conducted a study using the Guo method on 330 PANs in 2021, they did not provide any information on rater agreements.

Our results for the inter-rater agreements were lower overall than the values published in the literature for the individual methods [36–39, 48, 49]. In contrast to all previous studies, our study evaluated the correlation between three independent examiners, which can explain the reduced agreements. A variety of factors must always be taken into account when assessing observer agreement between different studies, including differences in sampled populations, imaging setup and quality, experimental conditions, and differences in raters. A strength of our study, however, is that evaluation across three independent examiners may offer a better estimate of the differences that might exist in real-world evaluations compared with the determination of the agreement of only two examiners.

Previous studies using the Olze method have examined far in excess of two thousand individuals in various populations [28, 36–39, 53, 54]. In contrast, the methods presented by Lucas et al. and Guo et al. are supported in particular by the studies of their first description [48, 49] or in single smaller studies [58].

## Conclusion

In our study, the staging method according to Olze et al. showed the highest values for the correlation with chronological age and examiner agreements. Although differences were small between methods, we argue that the Olze method should be preferred for future studies, since this method currently has the most reference data available in the literature. However, whenever the inter-radicular periodontal ligament is not evaluable, the use of the Guo method could be considered.

## Data Availability

The datasets generated during the current study are available from the corresponding author on reasonable request.
